# Diagnosing the ability of reservoir operations to meet hydropower production and fisheries needs under climate change in a western cordillera drainage basin

**DOI:** 10.1007/s10584-023-03632-y

**Published:** 2023-11-21

**Authors:** Samah Larabi, Markus A. Schnorbus, Francis Zwiers

**Affiliations:** Pacific Climate Impacts Consortium, University of Victoria, Victoria, BC Canada

**Keywords:** Water temperature, Water regulation, Climate Change, Nechako Reservoir

## Abstract

Water regulation has contributed to the decline in Pacific salmon in British Columbia (Canada) despite attempts to manage reservoir operations to achieve operational requirements while meeting environmental needs to limit fish thermal stress. The ability of reservoir managers to meet these trade-offs in a changing climate is unknown. Here, we examine the reliability and vulnerability of the Nechako Reservoir to meet hydropower production commitments and fisheries needs under two projected Shared Socioeconomic Pathway scenarios (SSP2-4.5 and SSP5-8.5). While our findings are specific to the operation of the Nechako Reservoir, the issues that emerge are likely common to many reservoirs in areas where reservoir inflow regimes are currently snow-storage dominated. We found that projected changes in the timing of water availability have little to no influence on hydropower generation commitments. However, larger water releases will be required to avoid compromising reservoir safety, possibly endangering downstream fish habitat through scouring. Furthermore, the temperature of water released from the reservoir is projected to more frequently exceed a level, 20°C, that is detrimental to migrating sockeye salmon. Water released is subject to further warming as it travels towards the lower reaches of the Nechako River used by migrating salmon. Hence, there is a need to adapt reservoir operations to ensure reservoir safety and mitigate adverse effects on salmon habitat.

**Supplementary Information:**

The online version contains supplementary material available at 10.1007/s10584-023-03632-y.

## Introduction

The Pacific Northwest (PNW) region of North America has extensive water resources infrastructure serving different purposes including hydropower generation, flood control and water supply (Hamlet 2011). This infrastructure induces water changes that threaten fish survival and fish habitat in downstream rivers. Of particular concern is the impact of dam regulation on water temperature that affects fish in many ways including growth/metabolic rate (Jonsson and Jonsson 2009; Beauregard et al. 2013), increased disease risk (Marcos-López et al. 2010), increased stress (Pérez-Casanova et al. 2008) and altered migration times (Mathes et al. 2010). This seems quite evident in British Columbia, Canada, which has seen a long term decrease in Pacific salmon populations (Price et al. 2017) due to the cumulative effects of dam regulation and resources exploitation in general (Chalifour et al. 2022). To mitigate the effects of dam regulation in rivers of the PNW, releases are often managed to maintain environmental flows that are designed to sustain riverine ecosystems and decrease stress on fish (e.g., Gu et al. 1999; Bradford et al. 2011).

Environmental flows (e-flows) aim to mimic natural flow regimes during key periods of the year so as to maintain a balance between ecosystem and human needs (Reidy Liermann et al. 2012). They are designed to provide the quantity of water required to maintain fish populations throughout their life cycles and/or to sustain other instream uses (EL-Jabi and Caissie 2019). Continuing climate change will affect water availability, thus e-flows that are designed to protect ecosystem and recreational values will need to be adapted to those changes. It is therefore necessary to evaluate current e-flow designs to understand their limitations under a changing climate and develop suitable adaptation strategies.

Projected changes in the PNW water cycle caused by continuing climate change include earlier snowmelt, higher winter rainfall, and lower summer flows (Wu et al. 2012). In addition, water temperatures will also increase, which will adversely affect Pacific salmon (Van Vliet et al. 2013). However, climate change impact on salmon survival may vary by location and species, and assessments of the combined effect of climate change and flow regulation on salmon have been few (Zhang et al. 2019), including the ability of e-flows to maintain environmental objectives, which remains untested. Although the projected changes in water timing and volume availability are similar over the entire PNW region, the effect of dams and reservoir operations are specific to each system. Nevertheless, the study of individual reservoirs can provide broader lessons for other parts of the region that motivate questions to consider when studying other reservoirs.

In this study, we consider the Nechako Reservoir, which was created in the 1950s to power an aluminum smelter at Kitimat on the British Columbia coast. By creating the reservoir, eastward-flowing water was redirected westward to the Kemano Powerhouse and then released to the Kemano River. Excess water is released mid-way via a spillway located at Skins Lake (the Skins Lake spillway, hereafter denoted SLS; see Section 2 for further details), which then flows to the Nechako River. This river is an important tributary of the Fraser River (the world’s most productive salmon river system; Northcote and Atagi 1997) and is of ecological and cultural significance to the Carrier (‘Dakelh’) First Nations which comprise 15 First Nations living within the Nechako watershed (CSTC 2005; Picketts et al. 2020). The impoundment of the Nechako Reservoir has negatively affected downstream river fish resources, particularly Chinook salmon, sockeye salmon, and endangered Nechako White sturgeon populations that have experienced long-term decreases (Gateuille et al. 2019). It also negatively affected the Nechako First Nations that rely on the river’s resources to sustain their way of life, particularly the Cheslatta T’En whose traditional lands were flooded (Windsor and Mcvey 2005) by the impoundment.

**Fig. 1:**
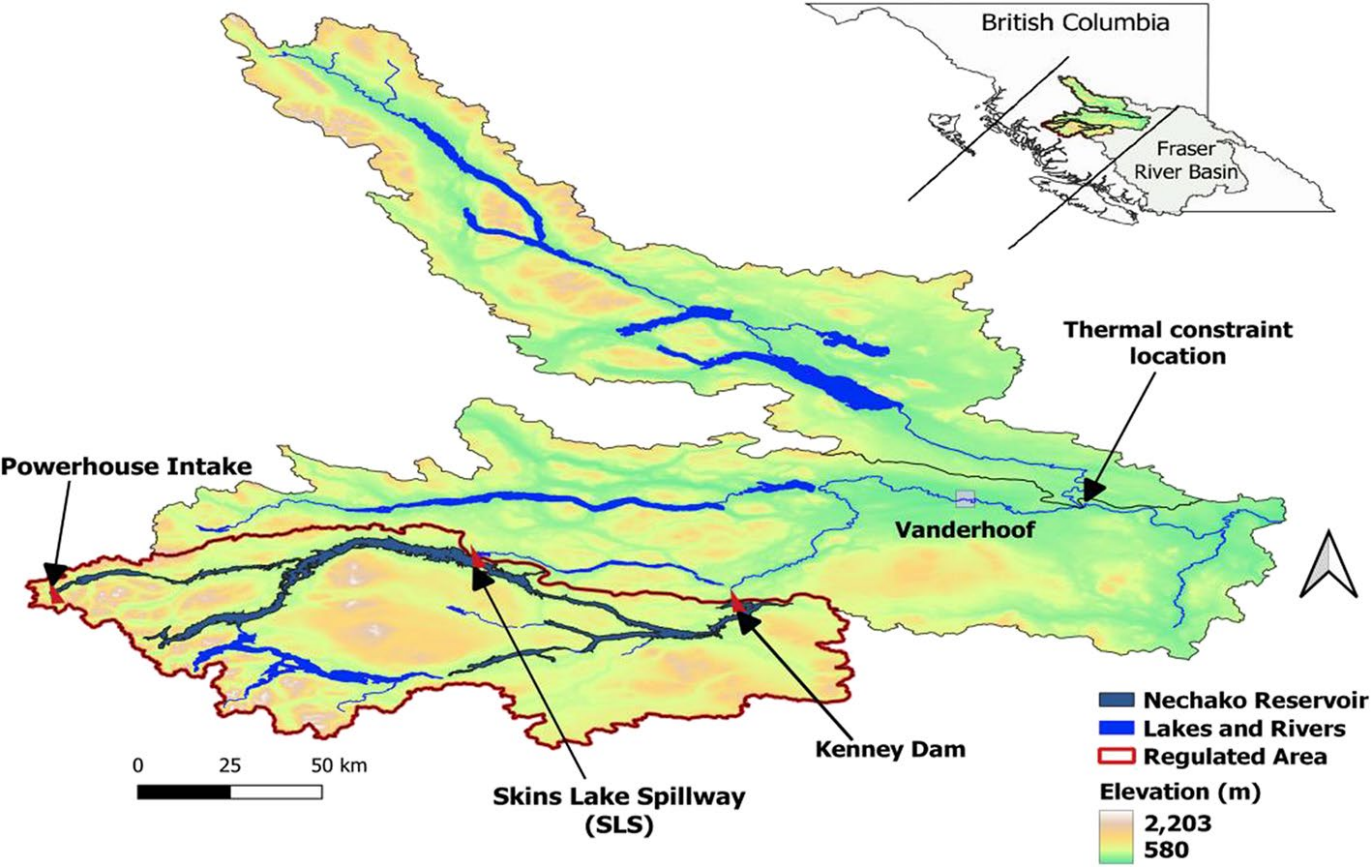
The Nechako Reservoir with its main outflow locations and the thermal constraint location at Finmoore

Concerns over the impact of the Nechako Reservoir on the river fish resources, led to the implementation of e-flows in the 1980’s to avoid dewatering conditions that affect Chinook salmon and to maintain daily average water temperature below 20 °C at Finmoore (thermal constraint location shown in Fig. 1) during sockeye migration between July 20th to August 20th (Macdonald et al. 2012). Average flow is maintained at a level of at least 32 m^3^ s^-1^ outside the July 20th to August 20th period, and is increased during this period to maintain water temperature below a daily average of 20 °C at Finmoore, which is well downstream of the SLS. E-flows released at SLS draw water from the reservoir’s epilimnion layer as it does not have facilities to draw water from deeper layers.

Historically, e-flows have effectively maintained water temperature below the critical 20 °C threshold (Macdonald et al. 2012; Fraser Basin Council 2015). Previous studies have analyzed the effect of climate change on water temperature in the Nechako River and along tributaries of the Fraser River basin in general (Islam et al. 2019). To our knowledge, however, no study has addressed the projected impact of climate change on the hydrology of the Nechako watershed above Kenney Dam and its impact on the reservoir’s ability to sustain trade-offs between hydropower generation, flood control and fisheries needs.

Accordingly, the purpose of this paper is to assess the response of the Nechako Reservoir to climate change under current reservoir rules. We use a holistic approach to assess the effect of climate change on the timing, volume, and temperature of reservoir inflows, and its impact on reservoir hydrodynamics and energy balance under current reservoir operating rules. The aim of such an approach is to understand limitations of current reservoir rules under climate change on hydropower generation, reservoir safety and the projected efficacy of current e-flows to provide cooling water to sockeye salmon during their migration period and adequate winter baseflow for Chinook survival. These limitations arise because the Nechako Reservoir has insufficient storage capacity to store inflows during high inflow years for use during dry years, and because the temperature of water released at the SLS depends on reservoir surface water temperature. The results of this study are intended to provide upstream boundary conditions for a subsequent study of the impacts of projected climate change on water temperature downstream of the reservoir under current operating rules given the system limitations.

## Study area

Kenney Dam was constructed in the early 1950s to create the Nechako Reservoir and regulate the Nechako River. The reservoir diverts water westward to the Kemano Powerhouse to generate power for an aluminum smelter at Kitimat on the British Columbia coast (see Fig. 1). Water that exceeds power requirements is released to the Nechako River through SLS.

**Fig. 2:**
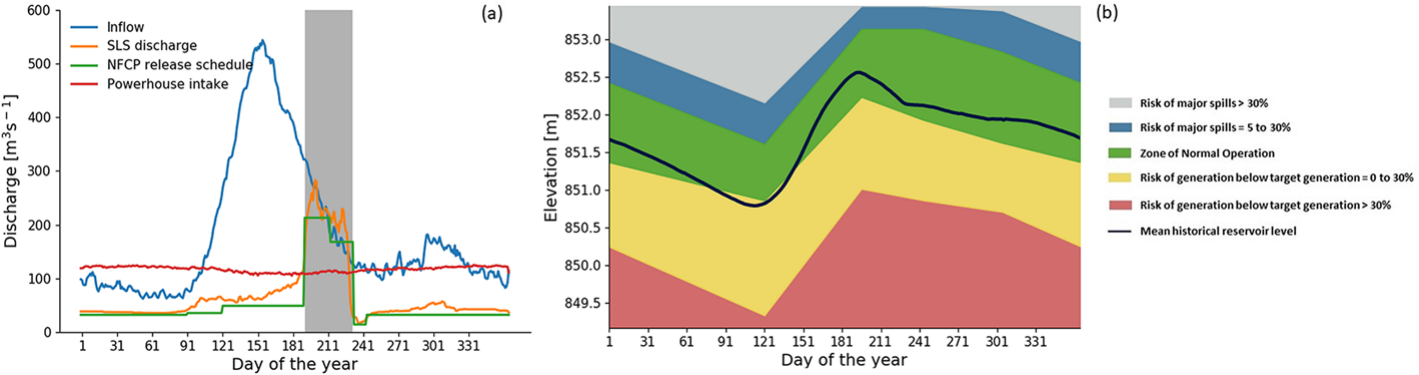
Daily Climatology of inflows and outflows with the shaded area representing the Summer Temperature Management Program (STMP) period (**a**) and reservoir levels with operational zones (**b**) of the Nechako Reservoir over the reference period 1981–2010. SLS discharge is the climatology of total discharge including e-flows and extra spills, and NFCP release schedule is the e-flows schedule as required by the Nechako Fisheries Conservation Program used in the study

The hydrologic regime of the Nechako Reservoir is typical of northern snow-dominated watersheds, where a large late spring or early summer snowmelt freshet provides most of the inflow and is the main source of reservoir refilling (Fig. 2a). Average powerhouse intake is around 117 m^3^ s to sustain continuous power generation. To reduce the risk of flooding, drawdown volumes are managed in advance of the annual freshet when the forecast inflow volume based on snowpack measurements is greater than the combined volume of storage available and the scheduled water volume to be released for Nechako Fisheries Conservation Program (NFCP) requirements and power generation (Rescan 1999).

The 1987 Settlement Agreement enforcing a flow management schedule for SLS discharge was implemented via the NFCP (NFCP 2016) (see Fig. 2a) following concerns over the negative effects of reservoir operations on Nechako River salmon populations. The main goals of the NFCP are 1) to ensure that flow changes do not endanger Chinook salmon and 2) to reduce water temperature below a daily average of 20 °C, the thermal tolerance threshold of sockeye salmon during their migration, at Finmoore (see thermal constraint location in Fig. 1). SLS discharge is therefore managed to meet the Annual Water Allocation and the Summer Temperature Management Program (STMP) requirements that are embodied within the NFCP. The Annual Water Allocation requirement ensures that a minimum average flow of 32 m^3^ s^-1^ is released through SLS throughout the year for the benefit of Chinook salmon. In addition, the STMP is applied between July 10th and August 20th to moderate water temperature during the control period July 20^th^ and August 20^th^ by manipulating SLS discharge timing and volume (Fig. 2a). STMP flow releases are based on 5-day meteorological forecasts (Triton 2021). Temperature control at the constraint location requires a 5-day lead in SLS releases (NFCP Technical Committee 2015). Between July 10^th^ and July 15^th^, water releases at SLS are increased to surcharge the Cheslatta system and maintain a minimum 170 m^3^ s^-1^ on July 15, after which flows are regulated according to predicted meteorological conditions. At the end of the control period water releases at SLS are dropped to 14.2 m^3^ s^-1^ to decrease flows at the Nechako River by early September to maintain fall spawning flows (Triton 2021). SLS releases are increased again in early September to the winter flow of 32 m^3^ s^-1^. SLS discharge is also increased when reservoir levels are above normal to ensure reservoir safety. Figure 2b, which is based on information provided by Rio Tinto, shows how the operational reservoir zones vary throughout the year. The normal operation zone represents the optimal reservoir level range where both hydropower generation and NFCP water release requirements are met. Reservoir levels below this zone are associated with a risk of below target power generation. Levels above this zone assure generation targets are met but imply a risk of higher than scheduled SLS releases.

## Methods

**Fig. 3:**
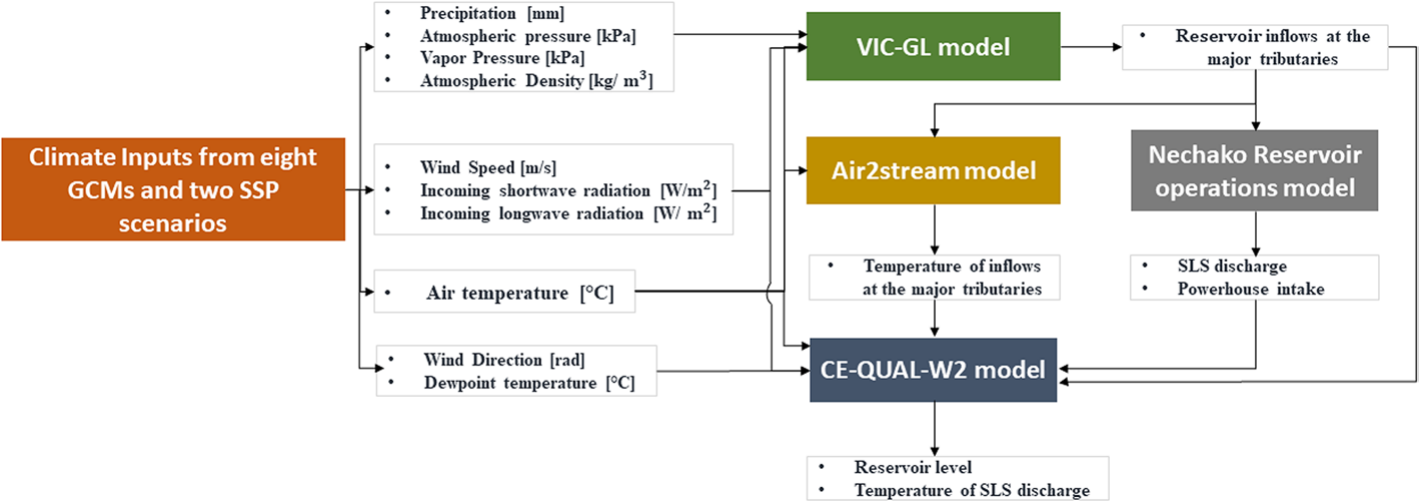
Schematic diagram of the model implemented to evaluate the response of the Nechako Reservoir watershed to climate change

A coupled hydrologic, stream temperature, reservoir operation, and hydrodynamic model was used to simulate the volume and temperature of inflow, powerhouse intake, thermal stratification and temperature of reservoir water, and the volume and temperature of water released. A schematic diagram of the modelling approach is depicted in Fig. 3. The approach is similar to that used by Larabi et al. (2022) to evaluate the characteristics of the Nechako Reservoir thermal stratification and temperature of water released at SLS. Here, we couple the hydrologic and water temperature model with a Nechako reservoir operations model to generate projections of reservoir outflows (i.e., SLS discharge and Powerhouse intake). Outputs from these models provide boundary conditions for the CE-QUAL-W2 model to simulate reservoir levels and temperature of SLS discharge. Results are obtained by driving the coupled models with climate scenarios derived from an ensemble of eight Global Climate Models (GCMs) from the Coupled Model Intercomparison Project Phase 6 (CMIP6) and two Shared Socioeconomic Pathway (SSP) scenarios (SSP5-8.5 and SSP2-4.5). SSP5-8.5 assumes an energy intensive, rapid growth economy based on fossil fuels and represents the high end of future forcing pathways, whereas SSP2-4.5 assumes a continuation of historical socioeconomic trends that produces intermediate levels of radiative forcing (O’Neill et al. 2016). We compare changes projected for two time frames: mid-century (2040–2069) and end- of-century (2070–2099) with a recent baseline period (1981–2010). Temperature changes are expressed in physical units as differences in °C between projected and baseline values while changes in water quantities (e.g., precipitation, inflow) are expressed in relative units as percentage change relative to baseline values. The projected change is evaluated for the eight CMIP6 models over the entire Nechako Reservoir watershed. Median projected changes are reported in the text, while figures show both medians and ranges.

### Modelling reservoir inflow volume and temperature

An upgraded version of the Variable infiltration Capacity (VIC) model (Liang et al. 1994, 1996, 1999), termed VIC-GL, was used to simulate reservoir inflows. VIC is a spatially distributed land surface model simulating both water and energy balances. VIC-GL (Schnorbus 2018) was implemented for each of the six sub-basins upstream of the Nechako Reservoir as identified by Water Survey Canada (WSC) hydrometric stations, namely Whitesail River near Ootsa Lake, Tahtsa River near Ootsa Lake, Eutsuk River at Outlet of Eustuk Lake, Entiako River near the Mouth, Chedakuz Creek at the Mouth, and Chelaslie River near the Mouth. Although additional historical WSC gauge stations are available, the delineation and calibration of the Nechako Reservoir was based on the mentioned six stations as they provide the best combination of continuous observed streamflow and water temperature data. For instance, the historical WSC gauge on the Tetachuk River only recorded seasonal data and the location has no observed temperature data. We opted instead to delineate the two headwater basins corresponding to the historical Tahtsa and Whitesail Rivers located farther upstream. Although these locations also do not have observed temperature data and only recorded seasonal streamflow, these outlet locations are located closer to the locations where contemporary water temperature observations have been collected (namely Coles and Laventie).

Delineation of the Nechako Reservoir basins based on these stations can be found in Larabi et al. (2022). Although VIC-GL is capable of modelling glacier mass balance and dynamics, glacier processes were not considered in the Nechako due to the negligible extent of glacier coverage (1.4% of the drainage area above SLS). VIC-GL was calibrated for each sub-basin by jointly maximizing the Nash Sutcliffe Efficiency (NSE, Nash and Sutcliffe 1970) and NSE of log daily streamflow (LNSE) to consider both low and high flows. The NSE of the calibrated model ranges between 0.42 (Entiako River near the Mouth) to 0.78 (Eutsuk River at Outlet of Eutsuk Lake) and the relative bias ranges between 1% (Eutsuk River at Outlet of Eutsuk Lake) to 16% (Whitesail River near the Ootsa Lake). Details of the calibrated model and its performance can be found in Larabi et al.(2022). Comparisons of simulated and observed streamflow timeseries are provided in supplementary Fig. 1S.

Water temperature in the six tributaries feeding the Nechako Reservoir was simulated with air2stream (Toffolon and Piccolroaz 2015), a hybrid physically based statistical stream temperature model. It simulates stream water temperature based on air temperature and discharge by considering the heat budget between an unknown volume of the river reach, the atmosphere, and the surface and subsurface water fluxes. It can be configured in multiple ways using parameterizations of varying complexity. Using VIC-GL simulated discharge, air2stream was tested at each site in 3-, 5- and 8-parameter configurations to identify the optimal version to use. Water temperature data (Triton 1994) are only available for WSC gauging stations at the outlets of the six basins for a short period (June to October 1994). Water temperatures are not available at the outlet of Whitesail River near Ootsa Lake and Tahtsa River near Ootsa Lake. Therefore, air2stream model was calibrated with water temperature recorded at nearby stations (Coles Creek above Troitsa Creek and Laventie Creek near the Mouth) for these two sub-basins. Air2stream was successfully calibrated using Particle Swarm Optimization (PSO, Kennedy and Eberhart 1995) to minimize the Root Mean Square Error (RMSE). The RMSE of the calibrated model ranges between 0.50 to 0.64 °C. Observed and simulated timeseries for summer 1994 are presented in Fig. 1S (supplementary material).

### Modelling reservoir operations

A Nechako Reservoir Operation Model provided by Rio Tinto was used to simulate reservoir operations. This model attempts to mimic day-to-day reservoir operations according to daily inflows (Boudreau 2005). Based on reservoir level and time of the year (see Fig. 2b), the model simulates the powerhouse intake and spillway releases under the following constraints. The reservoir level should be maintained above the minimum operational water level (849.5 m) to satisfy powerhouse load and generation commitments and below the maximum operational water level (853.44 m) to avoid dam breaches. The constraints also include the hydraulic characteristics and limitations of the system (i.e., maximum possible generation and maximum water release through the gates) as well as the NFCP e-flows schedule (Fig. 2b). Average flows at the spillway are maintained at 32 m^3^ s^-1^ from early September to late April and are increased to 213 m^3^ s^-1^ during the STMP period. To mitigate flood risk, flood releases are scheduled in advance of the spring freshet by relying on a forecast of May to August inflow volume (May 1st is assumed to mark the start of the snowmelt freshet; Boudreau 2005). However, for simulation purposes, the reservoir model is configured to “look ahead” by using VIG-GL simulated May to August inflow for the current season in place of a forecast (that is, for simulation purposes, we have assumed perfect inflow forecast skill). SLS discharge is increased to prevent flooding when the forecasted inflow volume is greater than the combined volume of storage available and scheduled generation and NFCP water releases. A description of the model, which was developed by Alcan (Rio Tinto’s predecessor), and was obtained from Rio Tinto by request, can be found in Boudreau (2005; Section 4.3).

### Modelling reservoir hydrodynamics

Reservoir and SLS discharge water temperature were simulated with the CE-QUAL-W2 model (see Larabi et al. 2022, for implementation details). CE-QUAL-W2 is a two-dimensional hydrodynamic and water quality model (Cole and Wells 2018) that requires bathymetry, inflow volume and temperature, outflow volume and meteorological data including air temperature, dew point temperature, wind speed and direction, and incoming solar radiation.

CE-QUAL-W2 simulated water surface elevations were validated by comparing simulated and observed values from 1986 to 2017. Model performance in simulating the reservoir thermal stratification was evaluated by comparing simulated and observed water temperature profiles measured at Kenney Dam and Natalkuz Lake. Recorded water temperature profiles were made available by Triton Environmental Consultants (Triton 1994). The model’s ability to simulate outflow water temperature at SLS was evaluated by comparing model values with observations recorded during summers of 2016 and 2017 provided by Rio Tinto.

### Climate change scenarios

Climate change projections needed to drive the coupled Nechako Reservoir modelling system were developed using an ensemble of eight global climate models from the Coupled Model Intercomparison Project Phase 6 (CMIP6) (see supplementary Table 1S for the list of climate models) that ran the SSP5-8.5 and SSP2-4.5 emission scenarios. These eight models provide all climate variables required by the Nechako reservoir modelling chain at a sub-daily timestep. Only the first member of the ensemble of climate simulations available from each climate model is used.

Several CMIP6 models warm more strongly than predecessor CMIP5 models (Tokarska et al. 2020). The choice of models was made to efficiently examine the responses to greenhouse gas forcing as represented by models in the central part of the equilibrium climate sensitivity (ECS) range (1.8 to 5.6 °C) of CMIP6 models (Tokarska et al. 2020). The ECS of six out of the eightCMIP6 models used here is in the range 2.6 °C and 4.3 °C (Table 1S).

Outputs of the CMIP6 multi-model ensemble used here were bias corrected with the multivariate bias correction algorithm (MBCn; Cannon 2018) using the fifth generation of the European Centre for Medium-Range Weather Forecasting (ECMWF) Reanalysis data (ERA5; C3S 2017) as training data. Sub-daily (3 hourly) surface air temperature, precipitation, dew point temperature, wind speed, incoming shortwave and longwave radiation, and surface air pressure were bias corrected at the GCM resolutions listed in Table 1S and then bilinearly interpolated to the 0.0625° (6 km) VIC-GL resolution. The remaining required modeling chain inputs (i.e., atmospheric density, vapor pressure and relative humidity) were estimated from the bias-corrected and interpolated data (i.e., air temperature and surface air pressure).

## Results

**Fig. 4:**
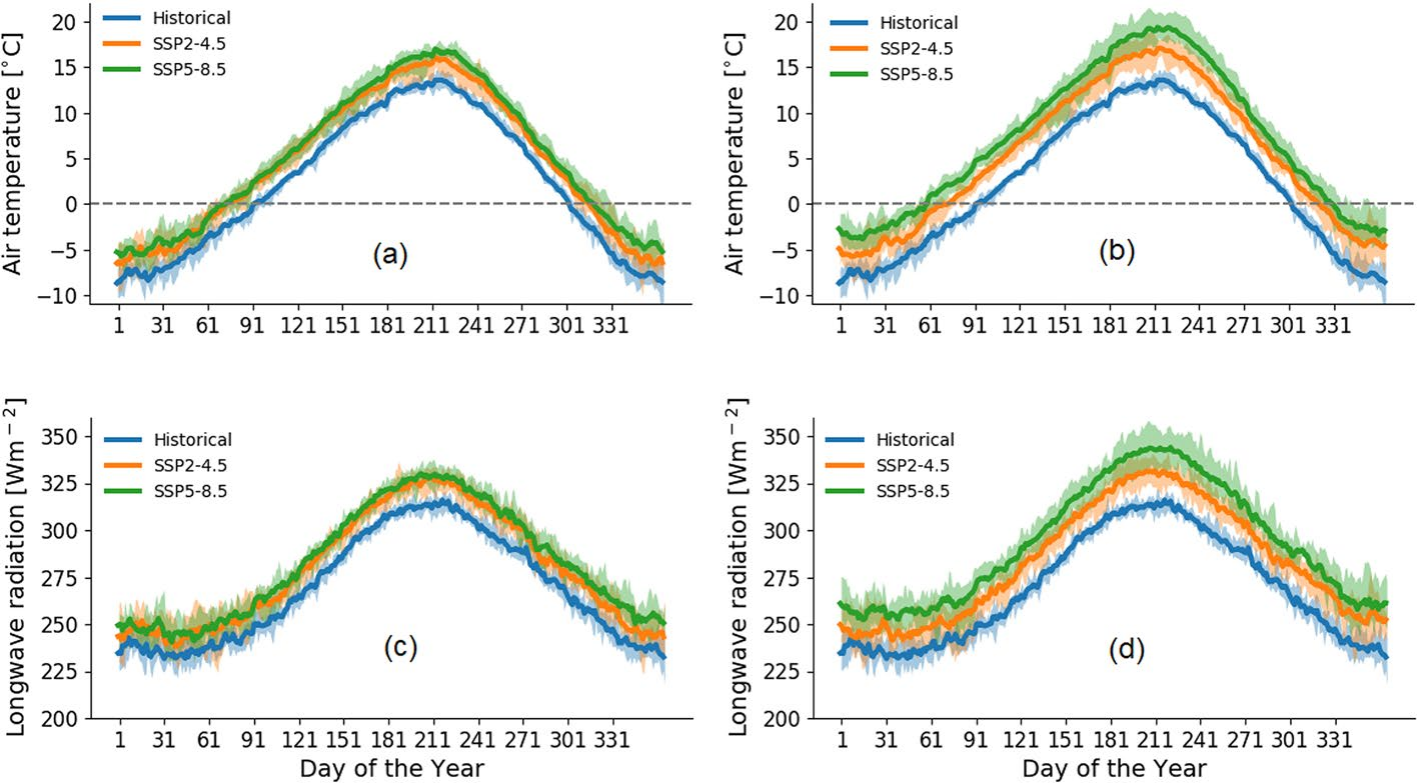
Baseline and projected daily climatology of air temperature and longwave radiation at mid-century (**a**, **c**) and end-of- century (**b**, **d**) with minimum and maximum range (colored shading) over the Nechako Reservoir basin

On an annual scale, air temperature over the modelling domain is projected to change by + 2.2 °C (mid-century under SSP2-4.5) to + 5.1 °C (end-of-century under SSP5-8.5). Total precipitation is projected to change by + 8% (mid-century under both emission scenarios) to + 14% (end-of-century under SSP5-8.5) while annual mean inflow is projected to change by + 5% (mid-century under SSP5-8.5) to + 10% (end-of-century under both emission scenarios), suggesting that increased evaporative demand may offset the larger precipitation increase under SSP5-8.5. On a monthly and seasonal scale, the CMIP6 multi-model ensemble projects an increase of air temperature year-around with a shorter season when air temperature is below freezing under both SSP scenarios and time horizons (Fig. 4a, b). The end-of-century monthly mean air temperature increase is more pronounced under SSP5-8.5 (+ 4.3 to + 5.5 °C) than under SSP2-4.5 (+ 2.3 to + 3.7 °C), particularly for summer months. Longwave radiation, which is important for snowmelt onset (Sicart et al. 2006) and is a dominant heat source to the reservoir (Larabi et al. 2022), is also projected to increase year-around under both emission scenarios (Fig. 4c, d). The eight CMIP6 models show similar monthly increasing patterns of longwave radiation under both emission scenarios at mid-century and exhibit end-of-century increases that could reach up to 15% under SSP5-8.5 (December).

**Fig. 5:**
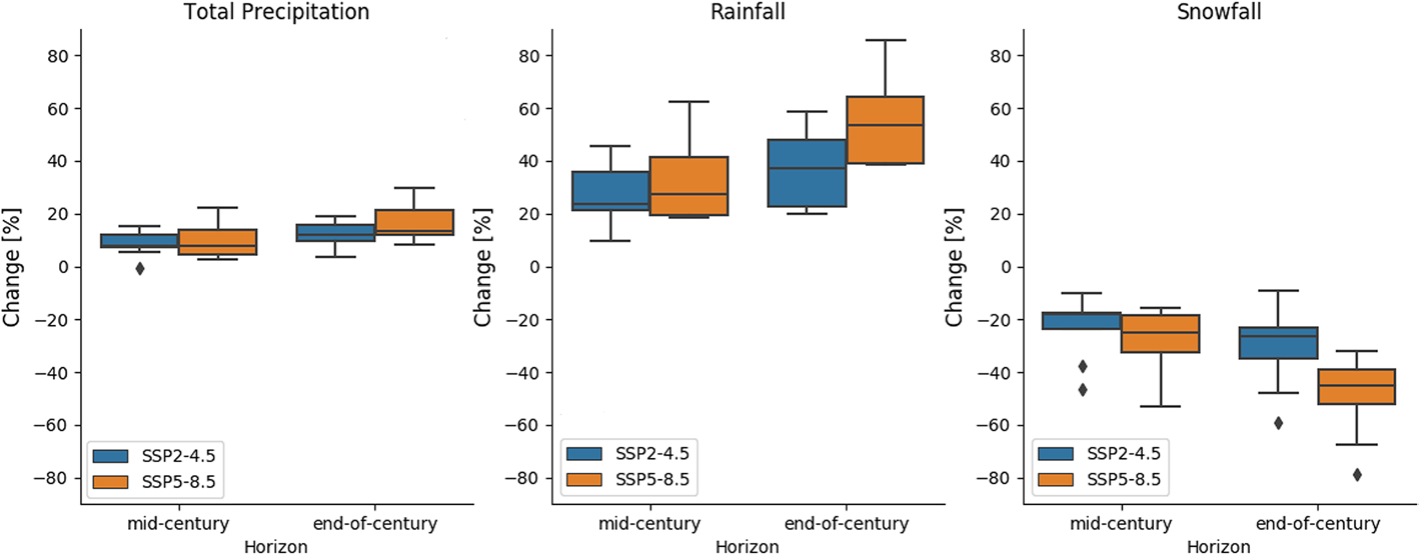
Distribution of projected relative change of total precipitation, rainfall and snowfall by the eight CMIP6 models. The bottom and the top of the boxes represent the interquartile range (25% and 75% quartiles). The bottom and top of whiskers represent 1.5 the lower and upper quartile range. The horizontal line inside the box represent the median of the ensemble and the dots are individual points that fall outside the whiskers ranges

With a shorter freezing period, annual total rainfall is projected to increase by 24% (mid-century under SSP2-4.5) to 54% (end-of-century under SSP5-8.5) and snowfall is projected to decline by 18% (mid-century under SSP2-4.5) to 45% (end-of-century under SSP5-8.5, Fig. 5). On a monthly scale, winter precipitation is projected to increase while summer precipitation is projected to decrease (Fig. 2S a,b,c, d) under both SSP scenarios with different magnitudes particularly at end- of-century. At mid-century, total monthly precipitation is projected to change by + 4 to + 14% under SSP2-4.5 and + 1 to + 18% under SSP5-8.5 with decreases of 2% and 6% respectively in the summer months. At end-of-century, SSP5-8.5 projects an annual mean precipitation increase of 3 to 31% whereas SSP2-4.5 projects a precipitation increase of 1 to 18%. Summer precipitation is projected to decrease by 12% and 6% under SSP5-8.5 and SSP2-4.5 respectively.

**Fig. 6:**
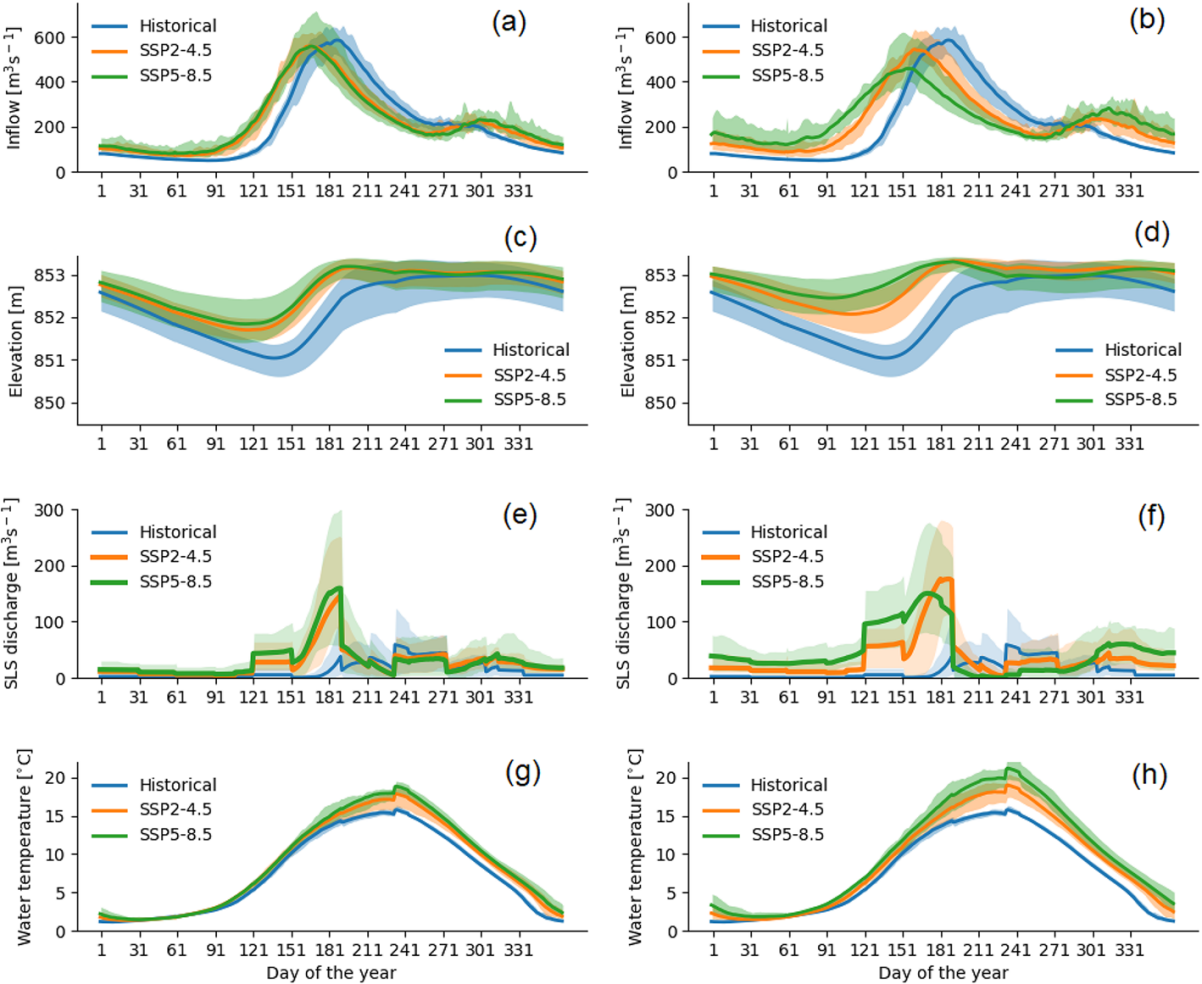
Projected daily climatology of reservoir inflow (**a**, **b**), reservoir levels (**c**, **d**), extra spills at Skins Lake Spillway (SLS; **e**, **f**) required to maintain reservoir level below the maximum operational level, and temperature of water released at SLS (**g**, **h**) by mid-century (left panel) and end-of-century (right panel) as simulated by the CMIP6 multi-ensemble model. Historical baseline values and the respective ensemble means are represented by the solid lines, and the ensemble range (minimum to maximum) is shown by the colored shading

All CMIP6 models agree on the projected pattern of monthly inflow changes (Fig. 6a, b) for both SSP scenarios and time horizons despite the lower level of consistency in projected monthly precipitation change between GCMs. Inflows are projected to increase during the cold period by 33% (November at mid-century under SSP2-4.5) to 166% (March at end-of-century under SSP5-8.5) and decrease during the warm period between July to September by 20% (September at mid- century under SSP2-4.5) to 44% (July at end-of-century under SSP5-8.5) (Fig. 2S. e, f, g, h). All models project the highest increase of inflow to occur in April, where change ranges between 125% (mid-century under SSP2-4.5) and ~ 320% (end-of-century under SSP5-8.5). Nevertheless, total annual reservoir inflow is projected to increase by only 5% (mid-century under SSP5-8.5) to 10% (end-of-century under SSP5-8.5 and SSP2-4.5). The monthly and seasonal inflow changes are substantial compared to the annual scale due to the freezing period shift, which causes an earlier but weaker freshet between March and June instead of April to August (Fig. 6a, b).

Using current reservoir rules, Nechako Reservoir levels are projected to frequently exceed historical reservoir levels in all months under both SSP scenarios over the century (Fig. 6c, d) due to increased annual inflow. The projected reservoir levels ensure continuous power generation, with little change in the mean average powerhouse intake. Reservoir level is projected to reach flood pool zones more often with the frequency of major spills (water released in addition to the SLS baseflow to prevent dam breaches) increasing to 5–30% by mid-century and to more than 30% by end-of-century (under SSP5-8.5 from March to July). As a consequence, the magnitude of water releases at SLS is projected to increase due to the requirement to maintain reservoir levels below the maximum operational level of 853.44 m. (Fig. 6e, f).

In the baseline period, simulated major spills occur between July and November with a maximum of 60 m^3^ s^-1^ in late August. At mid-century, major spills are projected to occur year-around with a maximum release in early July of ~ 150 m^3^ s^-1^ under SSP2-4.5 and 160 m^3^ s^-1^ under SSP5-8.5 (Fig. 6e). At end-of-century, SSP5-8.5 also projects an increase of major spill volume during winter as well as during the spring/summer freshet (Fig. 6f). Minimum flow requirements at SLS are projected to be satisfied at all times over the century under both emission scenarios. Total water releases at SLS are provided in supplementary material (Fig. 3S).

**Fig. 7:**
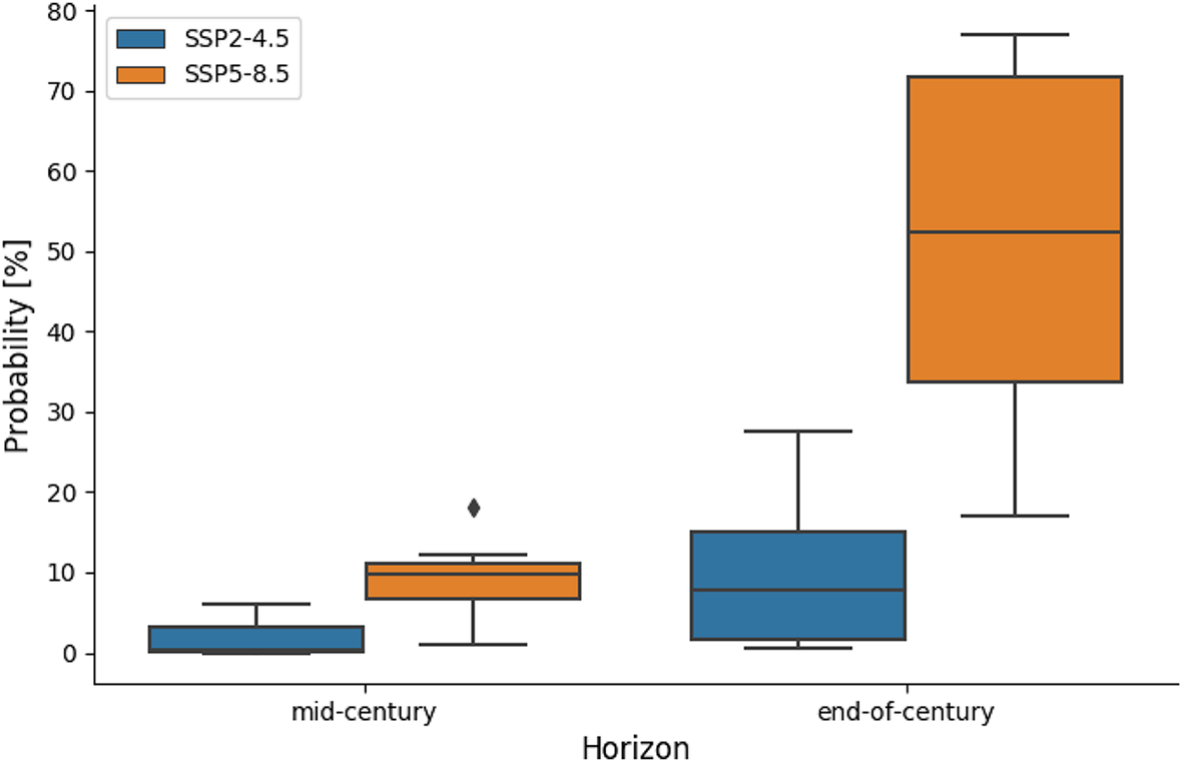
Distribution of the projected probability of outflow temperature at Skins Lake Spillway (SLS) exceeding 20 °C during the Summer Temperature Management Program period (i.e., sockeye migration period from 20th July to 20th August) simulated by the CMIP6 mutli-ensemble model. Probability of simulated SLS water temperature exceeding 20 °C during baseline period is 0. The bottom and the top of the boxes represent the interquartile range (25% and 75% quartiles). The bottom and top of whiskers represent 1.5 the lower and upper quartile range. The horizontal line inside the box represent the median of the ensemble and the dots are individual points that fall outside the whiskers ranges

Although both SSP scenarios project increasing SLS discharge temperature, magnitudes differ (Fig. 6g, h). Water temperature is projected to increase during most months of the year (except February and March) with the highest increases expected during July through September at mid- and end-of-century, particularly under the high-emission scenario. The summer water temperature (July–September) increase reflects the combined effect of increased air temperature and incoming longwave radiation, an earlier spring freshet, and decreased summer inflow. Air temperature and incoming longwave radiation are important heat sources to the reservoir surface layer from which SLS water releases are drawn (Larabi et al. 2022). During the baseline period, the simulated temperature of the SLS discharge never exceeds 20 °C during the annual STMP period. However, future projections show an increased probability of temperature exceeding 20 °C (Fig. 7). This exceedance probability remains very low under the SSP2-4.5 at mid-century (0 to 6%) but increases at end-of-century (1 to 28%). SSP5-8.5 projects a higher exceedance probability, particularly at end-of-century with a median value of 52% (17–77%, min–max) compared to mid-century (1 to 18%). The step change in water temperature observed in early September is related to the sudden increase in spillway discharge. This can be explained by the geometry of the spillway gates, which are two 11 m by 11 m radial gates that open upward from a sill at 842.77 m to a short free-crest at 853.44 m (Lawton 1953), and are represented as a single gate in CE-QUAL-W2 (Larabi et al. 2022). The water temperature gradient at the surface tends to be quite steep at the start of September (Larabi et al. 2022) such that at low discharge, flow is drawn from cooler sub-surface layers, whereas when the gate is more fully open, discharge also incorporates warmer surface water. Hence, the transition to higher releases in September has a warming effect.

## Discussion

**Fig. 8:**
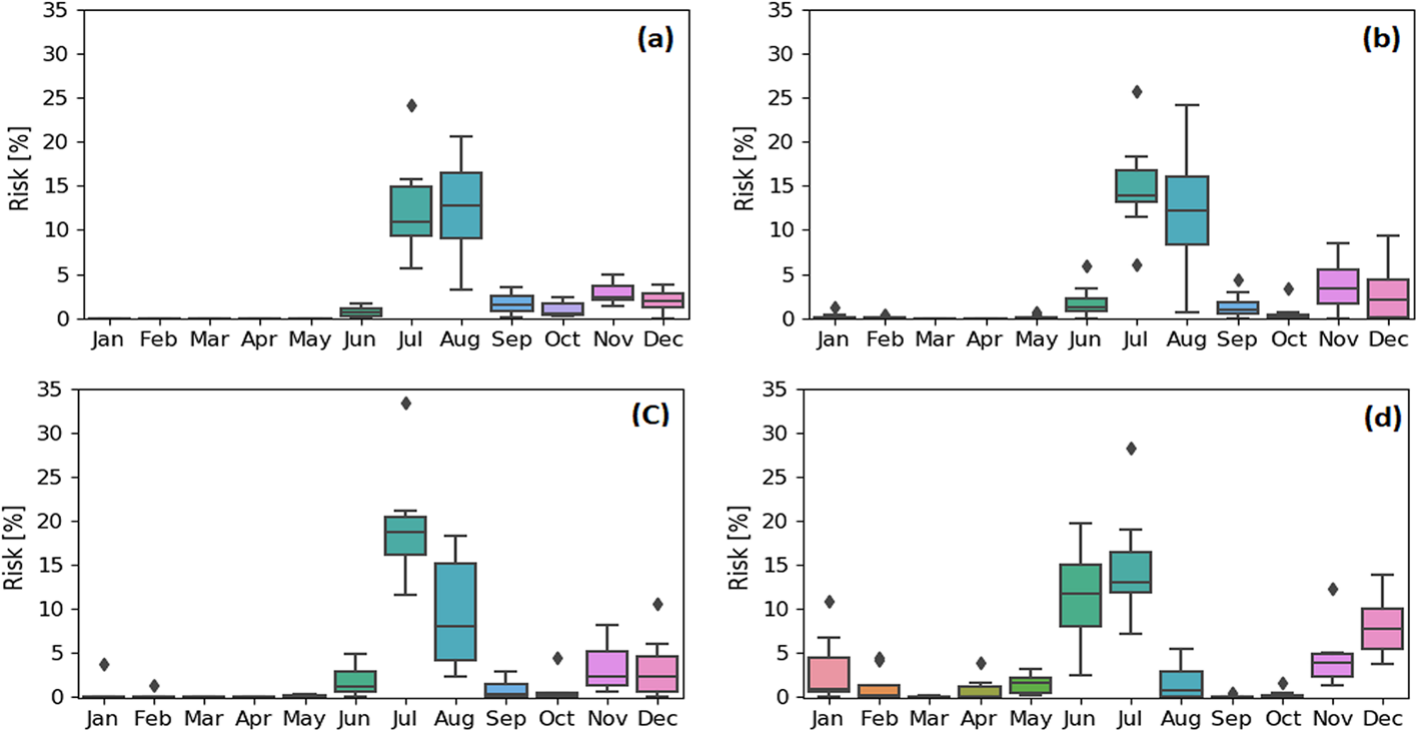
Projected risk of reaching maximum reservoir level (i.e., 853.44 m) by the eight CMIP6 models under SSP2-4.5 by mid-century (**a**) and end-of-century (**c**) and under SSP5-8.5 by mid-century (**b**) and end-of-century (**d**). The bottom and top of the whiskers represent the minimum and maximum ranges. The bottom and the top of the boxes represent the interquartile range (25% and 75% quartiles).The bottom and top of whiskers represent 1.5 the lower and upper quartile range. The horizontal line inside the box represent the median of the ensemble and the dots are individual points that fall outside the whiskers ranges

To mitigate flow risk, the timing and volume of reservoir releases must be adapted to consider changes to inflow seasonality. Reservoir management in snow-dominated regions leverages the strong seasonality of inflow and relies on snowpack-based forecasts to optimize drawdown volumes to reduce flood risks (Arsenault et al. 2016). Flood releases in the Nechako Reservoir are scheduled in advance of the annual freshet when the forecast volume of inflow from measured snowpacks is greater than the combined volume of storage available and the volume of water scheduled to be released for NFCP requirements and power generation (Rescan 1999). This study assumes perfect inflow forecasts by using model simulated water volume during the current warm season (i.e., historic snowmelt freshet season of May to August) as a forecast proxy. Under historical conditions when snowmelt is the main reservoir recharge source, current reservoir rules efficiently limit the risk of reaching the maximum reservoir level, with the probability of exceeding that level being under 9%. Under climate change, inflow seasonality is projected to shift from one dominated by spring snowmelt to a hybrid regime (inflow seasonality is affected by both fall/winter rainfall and spring snowmelt) by the end of the century (Fig. 6). Consequently, the seasonality of reservoir recharge is projected to shift and managing drawdown volumes based only on spring–summer snowmelt, as is done in our simulations, increases the risk of reaching maximum reservoir levels to 19% (under SSP2-4.5) (Fig. 8). Scheduling releases in this way under climate change also produces an increasing trend in reservoir storage due to increased autumn and winter runoff (Fig. 6).

In snow-dominated regions, snowpacks sustain summer inflows and serve as the main source of annual reservoir recharge. This seasonal inflow has high predictability, as its volume is largely determined by the volume of water in the snowpack (Shukla and Lettenmaier 2011; Shrestha et al. 2015). Consequently, snowpack-based forecasts have high skill and are foundational for reliable reservoir operations in the Western Cordillera (Wood et al. 2016). However, the decline of the snowpack and a shift to proportionately more inflow from fall-winter rainfall and transient snowmelt events will degrade the skill of snowpack-based inflow forecasts (Livneh and Badger 2020; Cohen et al. 2020; Shrestha et al. 2015). Increased fall/winter rainfall will also require more immediate water release decisions to prevent flooding, particularly for a reservoir with limited storage capacity like the Nechako Reservoir. In this study, the reservoir operation model assumes a perfect forecast skill of summer inflows. As inflows becomes less predictable under a hybrid inflow regime, the potential impact of forecast uncertainty on reservoir operations should be addressed to evaluate adaptation strategies.

**Fig. 9:**
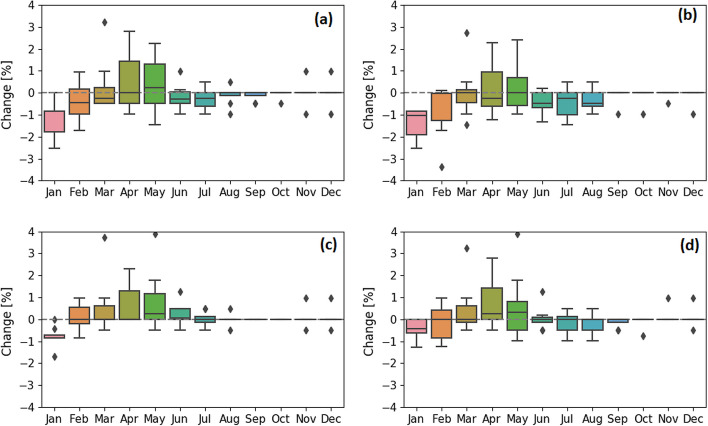
Projected change of hydropower production by the eight CMIP6 models under SSP2-4.5 by mid-century (**a**) and end- of-century (**c**) and under SSP5-8.5 by mid-century (**b**) and end-of-century (**d**). The bottom and the top of the boxes represent the interquartile range (25% and 75% quartiles). The bottom and top of whiskers represent 1.5 the lower and upper quartile range. The horizontal line inside the box represent the median of the ensemble and the dots are individual points that fall outside the whiskers ranges

Under historical conditions, the reservoir can reliably provide continuous power generation at all times. The system is projected to continue to reliably satisfy power generation commitments over the century under both SSP2-4.5 and SSP5-8.5 on an annual basis. Nevertheless, our modelling suggests that there may be a modest redistribution of power generation potential during the January to August period. By mid-century, both emission scenarios project a decrease of monthly hydropower production during January and February (Fig. 9a, b). This decrease could be a result of larger SLS water releases during previous fall months together with a significant proportion of precipitation continuing to fall as snow during the January–February period by mid-century. As snowfall declines and winter rainfall increases by end-of-century, particularly under SSP5-8.5, the magnitude of hydropower change during January decreases. Both emission scenarios project an increase of monthly hydropower production during April and May due to earlier water availability.

Due to limited storage capacity, average annual SLS discharge is projected to increase by 12% (mid-century under SSP5-8.5) to 27% (end-of-century under SSP5-8.5). The projected winter SLS discharge increase is higher than the annual average change (Fig. 6e, f), with increases ranging from 18% (mid-century under SSP2-4.5) to 43% (end-of-century under SSP5-8.5). The potential for increased SLS discharge throughout the year suggests reservoir operations will need to adapt not only to ensure reservoir safety but also to manage the potential adverse impact of high peak flows. Larger water releases in winter could exacerbate downstream flooding events due to the occurrence of ice jams (Albers et al. 2016). Higher SLS discharge and possibly higher flow variability also has the potential to decrease egg-to-fry survival through scour of spawning nests (i.e., mobilization of sediment and lowering of streambed) (Healy 2011; Ward et al. 2015; Malcolm et al. 2012; Gendaszek et al. 2017), particularly for Chinook salmon. The median flow variability at SLS during fall/winter is projected to increase up to 45% (December under SSP5-8.5) by mid-century and up to 132% (December under SSP5-8.5). [See Fig. 4S in the supplementary material.] In the Nechako River, sediment transportation and deposition influenced by large SLS water releases have been identified as potential factors leading to the decline of Chinook salmon, sockeye salmon and endangered Nechako White sturgeon (Gateuille et al. 2019).

The Summer Temperature Management Program (STMP) was adopted to limit the occurrence of high water temperatures in the Nechako River at the thermal constraint location (approximately 240 km downstream of the SLS) during the sockeye salmon migration period by controling the timing and volume of SLS discharge (NFCP 2016). The STMP has been effective in reducing the occurrence of water temperature above 20 °C at the Nechako River at Finmoore (Macdonald et al. 2012; Fraser Basin Council 2015). However, future climate scenarios project substantial increases in summer water temperature and in the frequency with which SLS discharge temperature exceeds 20 °C. The water released at SLS is also drawn from the epilimnion layer, which is most sensitive to changes in meteorological forcing (Larabi et al. 2022), and cannot be adjusted to draw cooler water from depth. While SLS water releases can reduce Nechako River water temperatures, discharge volume adjustments that are possible within the range that is feasible for this reservoir and its spillway infrastructure are unlikely to be able to compensate for rising discharge temperature. Hence, it should be anticipated that the effectiveness of the STMP will be reduced in the future assuming no change in the existing spillway infrastructure. Given limited flexibility in altering discharge volume, and the fact that water discharged at the SLS is further heated as it flows downstream to the control point at Finmoore, BC, water discharged at the SLS in the future would have to be substantially cooler than the water that is discharged under today’s climatic conditions to reliably achieve the objectives of the STMP.

Results of this study suggest that alternative e-flows at SLS should be explored to potentially reduce the anticipated impact of climate change on water temperature downstream of the Nechako Reservoir. The current STMP takes into consideration only two salmonid species, i.e., sockeye salmon and Chinook salmon. However, the Nechako white sturgeon is another endangered fish population that is vulnerable to water temperature above 18 °C that should be considered in exploring new e-flows (Earhart et al. 2023). Climate change is also likely to affect fish species residing in the reservoir. Around 13 resident fish populations inhabit different depths of the Nechako Reservoir (Triton 2005). Impacts of projected changes in the reservoir thermal stratification on these species should be addressed particularly for species that are of importance to the Nechako Reservoir recreational fishing.

The findings of this study reveal the potential impact of climate change on the Nechako Reservoir assuming current operating rules at the SLS and water intake volume at the powerhouse consistent with historical powerhouse operations. The findings do not take into account the possibility of changes to reservoir operating rules, which could change for a variety of reasons. For example, day-to-day reservoir operations could change as a consequence of changes in snowpack forecasting or the adoption of additional flow targets in the Nechako River for enhanced flood mitigation and ice jam management (Rio Tinto 2019). These flow targets potentially include limits of 330 m^3^ s^-1^ at Cheslatta Falls to prevent flooding of ancestral burial grounds along Cheslatta Lake and 550 m^3^ s^-1^ to prevent flooding of the Nechako River at Vanderhoof. They could also include a 100 m^3^ s^-1^ limit in the Nechako River at Vanderhoof during the freeze-up period and a restriction on daily SLS discharge increases to no more than 15 m^3^ s^-1^ after freeze-up to avoid ice jam formation and maintain a trade-off between ice jam and flood risk in spring. Assessment of the reservoir to meet these trade-offs would require modelling the unregulated areas that drain to the Nechako River at Vanderhoof.

## Conclusions

It is well established that water regulation impacts downstream river fish habitats. To counter the negative effects of regulation, water management often includes measures to sustain environmental flow (e-flows). However, the future efficiency of e-flows under climate change is unknown. The impacts of projected changes in the water cycle on the efficiency of e-flows and reservoir operations and their subsequent impact on downstream fish habitat are specific to each system. In this study, we consider the Nechako Reservoir, which regulates the Nechako River, an important, culturally significant, migration corridor for salmon. We evaluated the effect of climate change on the ability of the reservoir to satisfy power generation commitments, the frequency and volume of water releases to limit flooding risk, and the future effectiveness of e-flows under current reservoir operation rules.

Our findings show that climate change has little to no influence on annual hydropower generation. Water used for hydropower generation is discharged to the Pacific Ocean, while all other discharges occur through a spillway that flows into a tributary of the Nechako River, and ultimately into the Fraser River. The limited storage capacity of the reservoir implies larger, and more frequent water releases will be required via the spillway to control flood risk and maintain reservoir levels below the maximum operating level. This change is due to the combined effect of a reduced snowpack and increased fall/winter rainfall. E-flows designed to limit water temperatures below the spillway during the late summer sockeye salmon migration period are also released via the spillway. As water that is released at the spillway is from the epilimnion, the ability to provide cooling water for migrating salmon during summer is projected to decrease because the temperature of the reservoir epilimnion layer is highly sensitive to increased air temperature and longwave radiation.

This study has evaluated climate change impacts on the Nechako Reservoir under current reservoir operating rules. These rules consider the hydraulic characteristics and limitations of the system as well as the water release schedule at Skins Lake Spillway as established by the Nechako Fisheries Conservation Program. Evaluation of different reservoir rules and e-flow designs, possibly considering withdrawal of reservoir bottom water, will be needed to better adapt water release schedules to alterations in water availability and improve the management of increased fall/winter streamflow to avoid any decrease in winter power generation. While our findings are specific to the operation of the Nechako Reservoir, the issues that emerge are likely common to many reservoirs in areas where reservoir inflow regimes are currently snow-storage dominated.

### Supplementary Information

Below is the link to the electronic supplementary material.

Supplementary file1 (DOCX 734 KB)

## Data Availability

This study uses a version of VIC4 that has been further developed at PCIC and coupled to a regional glacier model to simulate streamflow. The modified VIC model code is available at https://github.com/mschnorb/VIC. The air2stream model for water temperature simulation developed by Toffolon and Piccolroaz (2015) is freely available at https://github.com/spiccolroaz/air2water. The Linux version of CE-QUAL-W2 used here is available at https://github.com/WQDSS/CE-QUAL-W2-Linux. The Nechako Reservoir operation model was provided by Rio Tinto. Outputs generated from this study are available upon request from authors.
