# Supplementary material for: Diagnosing the ability of reservoir operations to meet hydropower production and fisheries needs under climate change in a western cordillera drainage basin
Source: Clim Change. 2023 Nov 21;176(12):161. doi: 10.1007/s10584-023-03632-y (PMC10663265; doi:10.1007/s10584-023-03632-y)
Supplement: Supplementary file 1 — Supplementary file1 (DOCX 734 KB) [file 10584_2023_3632_MOESM1_ESM.docx]

Diagnosing the ability of reservoir operations to meet hydropower production and fisheries needs under climate change in a Western Cordillera drainage basin

**Supplementary material**

1. **Performance of VIC-GL and air2stream**

Details of VIC-GL implementation to the Nechako Reservoir watershed can be found in Larabi et al., (2022). Simulated streamflow at the six major tributaries feeding the reservoir were supplied to the air2stream model (Toffolon & Piccolroaz, 2015). The air2stream model was calibrated against water temperature recorded in summer 1994 at the major tributaries: Chedakuz Creek (CHEDA), Entiako River (ENTIA), Chelaslie River (CHELA), and Eutsuk River at the Outlet of Eutsuk Lake (EUTSU). Water temperature is not available for Whitesail River near Ootsa Lake (WHITE) and Tahtsa River near Ootsa Lake (TAHOL). Therefore, we use water temperature records from nearby stations (Coles Creek above Troitsa Creek and Laventie Creek near the Mouth) to calibrate air2stream model for these sub-basins.


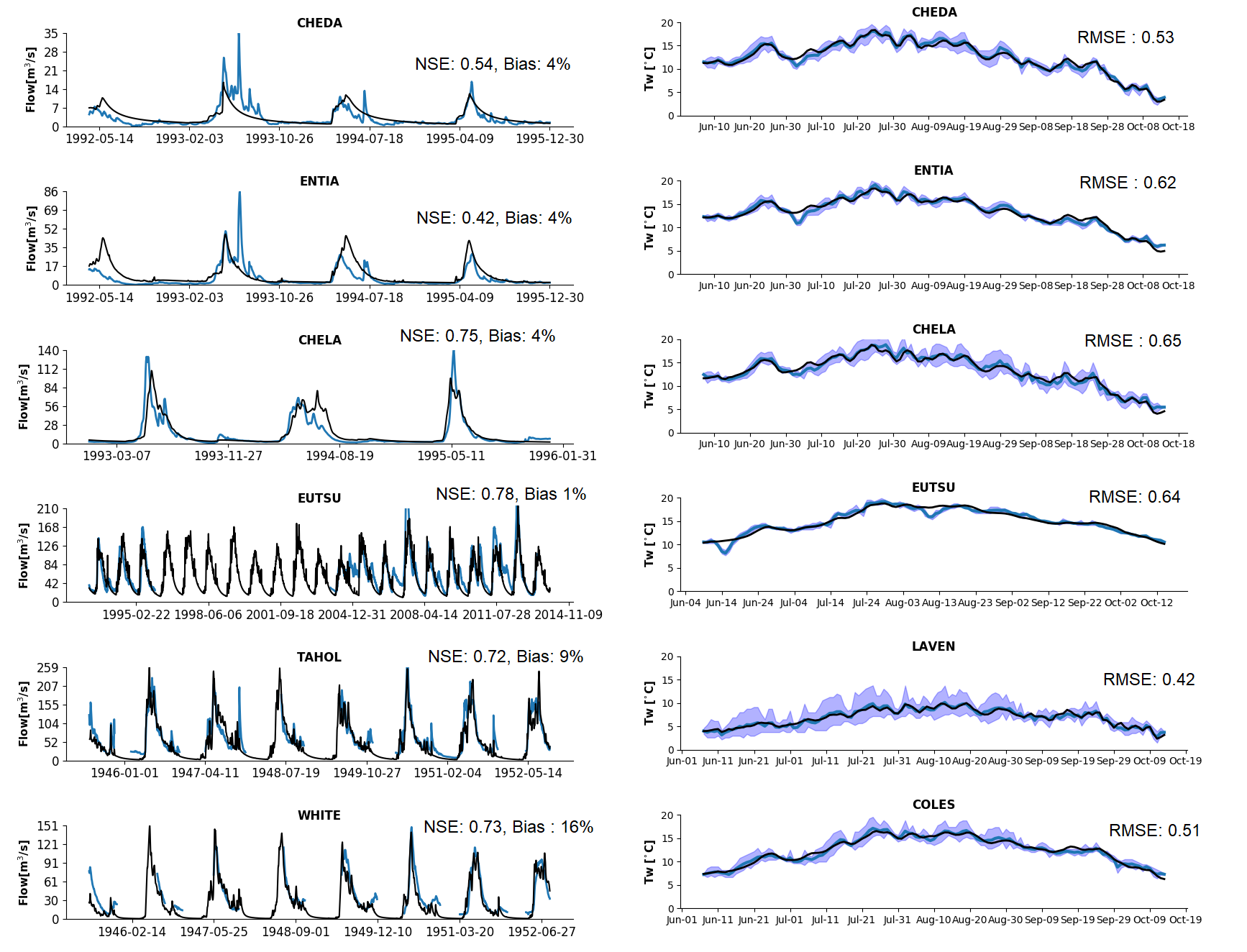


Figure 1: Observed (blue) and simulated (black) streamflow during calibration period (left panel), daily simulated (black) and observed water temperature (blue) with daily minimum and maximum values (ribbon) (right panel) at the Nechako reservoir upstream tributaries.

1. **List of CMIP6 models used**

**Table 1: The eight models of the Coupled Model Intercomparison Project Phase 6 (CMIP6). Equilibrium Climate Sensitivity (ECS) values are taken from Tokarska et al. (2020), Schlund et al. (2020) and Lovato et al. (2022).**

| **ID** | **MODEL** | **Resolution** | **ECS [°C**] |
| --- | --- | --- | --- |
| **1** | BCC-CSM2-MR | 100 km | 3.0 |
| **2** | CMCC-CM2-SR5 | 100 km | 3.5 |
| **3** | CMCC-ESM2 | 100 km | 3.6 |
| **4** | EC-Earth3 | 100 km | 4.3 |
| **5** | MIROC6 | 250 km | 2.6 |
| **6** | MPI-ESM1-2-HR | 100 km | 3.0 |
| **7** | MPI-ESM1-2-LR | 250 km | 3.0 |
| **8** | MRI-ESM2-0 | 100 km | 3.1 |

1. **Projected changes in monthly precipitation and inflows**


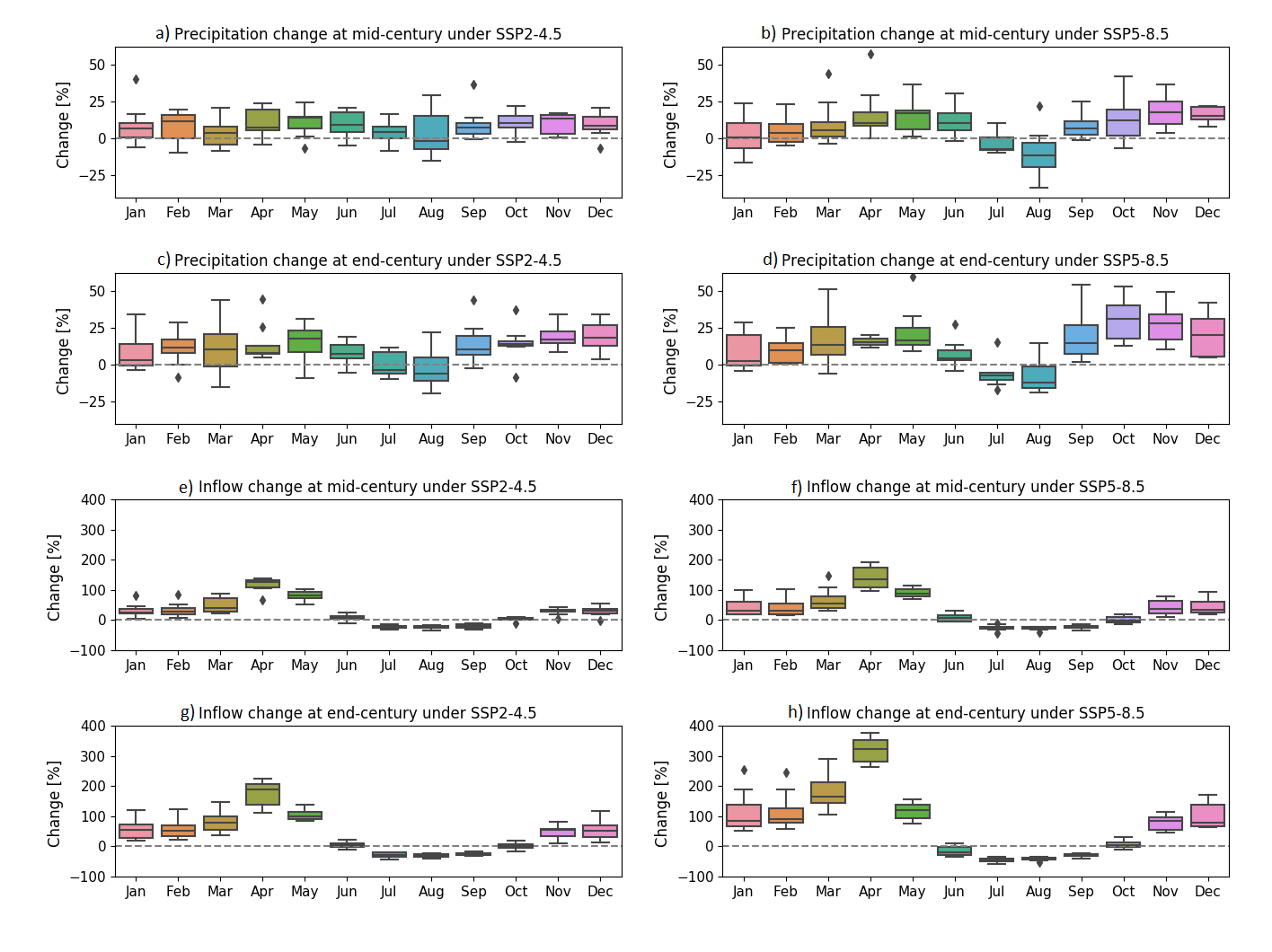


Figure 2: Monthly projected change in precipitation and inflow from eight CMIP6 models under the high and mid emission scenarios at mid century and end century. The bottom and top of the whiskers represent the minimum and maximum ranges. The bottom and the top of the boxes represent the interquartile range (25% and 75% quartiles). The bottom and top of whiskers represent 1.5 the lower and upper quartile range. The horizontal line inside the box represent the median of the ensemble and the dots are individual points that fall outside the whiskers ranges.

1. **Projected climatology of total discharge at Skins Lake Spillway**

Figure 3 presents the projected climatology of total water release at Skins Lake Spillway considering the limitations (minimum and maximum operation levels) and hydraulic characteristics (maximum possible generation and maximum water release through the gates) of the system, satisfaction of the powerhouse load and generation commitments and NFCP requirements. The NFCP requirements include annual water allocation requirements that ensures a minimum of average flow of 32 m^3^s^-1^ is released through SLS throughout the year for the benefit of Chinook salmon. It also includes the Summer Temperature Management Program (STMP) applied between July 10^th^ and August 20^th^ to moderate water temperature during the control period July 20^th^ and August 20^th^ by manipulating SLS discharge timing and volume. At the end of the control period water releases at SLS are dropped to 14.2 m^3^s^-1^ to decrease flows at the Nechako River by early September to maintain fall spawning flows (Triton, 2021). SLS releases are increased again in early September to the winter flow of 32 m^3^s^-1^.

Operationally, the STMP flow releases are based on 5-day meteorological forecasts during the control period (Triton, 2021). Between July 10^th^ and July 15^th^, water releases at SLS are increased to surcharge the Cheslatta system and maintain a minimum 170 m^3^s^-1^on July 15, after which flows are regulated according to predicted meteorological conditions. In response to warming trends, the SLS release criteria include increasing releases to 453 m^3^s^-1^ to achieve flow changes quickly at the Nechako River. Although the SLS releases in this study are not based on 5-day meteorological forecasts during the STMP period, they still reflect the main rules used for reservoir management.


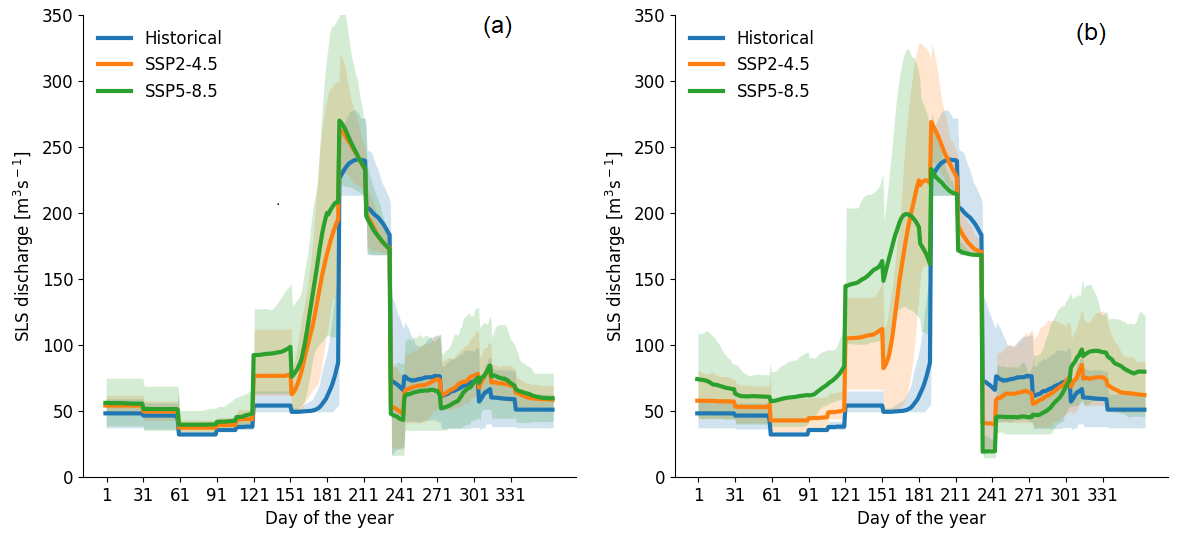


Figure 3: Baseline and projected daily climatology of total discharge at Skins Lake Spillway at mid-century (a) and end-of-century (b) with minimum and maximum range (colored shading).

1. **Monthly changes in flow variability at Skins Lake Spillway**


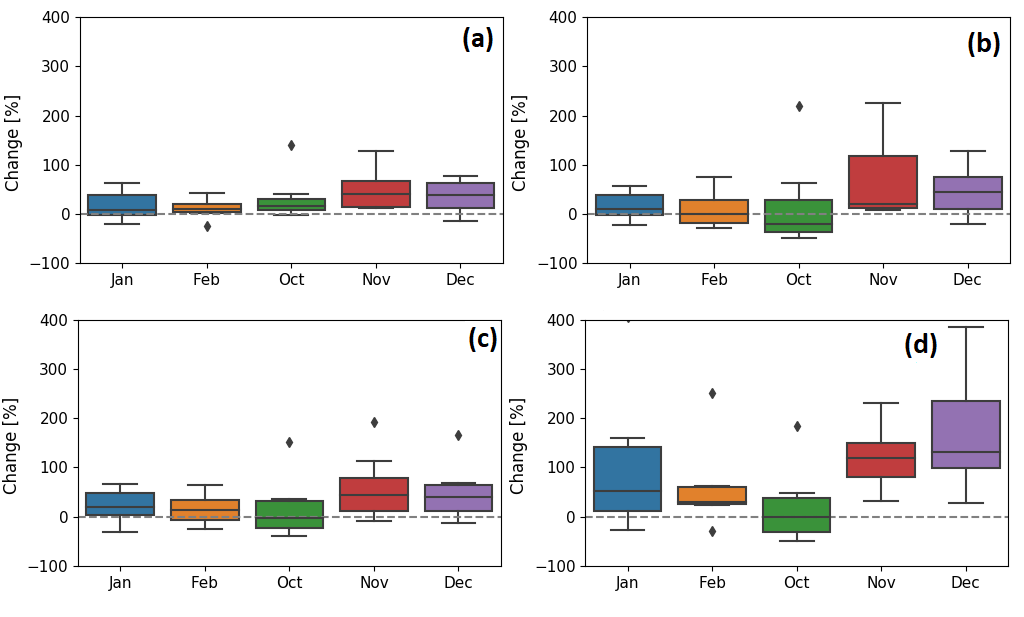


Figure 4: Projected change of flow variability during earliest life stages of Chinook salmon (October to February) by the eight CMIP6 models under SSP2-4.5 by mid-century (a) and end-century (c) and under SSP5-8.5 by mid-century (b) and end-century (d). The bottom and the top of the boxes represent the interquartile range (25% and 75% quartiles). The bottom and top of whiskers represent 1.5 the lower and upper quartile range. The horizontal line inside the box represent the median of the ensemble and the dots are individual points that fall outside the whiskers ranges.

**References**

Larabi, S., Schnorbus, M., Zwiers, F (2022) A coupled streamflow and water temperature (VIC-RBM-CE-QUAL-W2) model for the Nechako Reservoir. *Journal of Hydrology*: *Regional Studies*, 44 ,101237. https://doi.org/10.1016/j.ejrh.2022.101237

Lovato, T., Peano, D., Butenschön, M., Materia, S., Iovino, D., Scoccimarro, E., et al (2022) CMIP6 simulations with the CMCC Earth System Model (CMCC-ESM2). *Journal of Advances in Modeling Earth Systems*, 14, e2021MS002814. <https://doi.org/10.1029/2021MS002814>

Schlund, M., Lauer, A., Gentine, P., Sherwood, S.C., Eyring, V (2020) Emergent constraints on equilibrium climate sensitivity in CMIP5: do they hold for CMIP6? *Earth System Dynamics*, 11, 1233–1258. https://doi.org/10.5194/esd-11-1233-2020

Toffolon, M., Piccolroaz, S (2015) A hybrid model for river water temperature as a function of air temperature and discharge. *Environmental Research Letters*, 10, 114011. http://dx.doi.org/10.1088/1748-9326/10/11/114011

Tokarska, K.B., Stolpe, M.B., Sippel, S., Fischer, E.M., Smith, C.J. et al (2020) Past warming trend constrains future warming in CMIP6 models. *Science Advances*, 6, eaaz9549. https://doi.org/10.1126/sciadv.aaz9549

Triton Environmental Consultants Ltd (2021) 2020 Summer Water Temperature and Flow Management Project. Nechako Fisheries Conservation Program. 44pp.
